# Georgia Lunatic Asylum

**Published:** 1846-02

**Authors:** 


### THE WESTERN JOURNAL.

New Series.—Vol. V.—No. II.

LOUISVILLE, FEBRUARY 1, 1846.

### GEORGIA LUNATIC ASYLUM.

We have looked through the report of Dr. David Cooper on the "Lunatic, Idiot, and Epileptic Asylum of the State of Georgia," and find in it much to interest the physician and philanthropist. This institution was opened on the first of November, 1842. It is capable, at present, of accommodating one hundred patients; but, when the buildings now in progress shall have been completed, will be spacious enough to receive five or six times that number. The Legislature of Georgia have appropriated, at different times, sums to the institution, amounting in all to $45,000. The State has the privilege of sending its insane paupers to the asylum, and persons who are able to pay are charged the very small sum of $100 per annum, "for board, medical attendance, clothing, &c." No institution in the world, of the same character, affords these accommodations at so low a price. Yet, from the report of the resident physician, we are fully persuaded that the accommodations will be of the best kind. The edifice is constructed upon the most approved model, and embraces every comfort which ingenuity has introduced into modern buildings, both as to warming, cleanliness, and comfort. The grounds pertaining to the institution are ample, and every facility for enjoying exercise in the open air, in riding, walking, or working in the farm and garden will be afforded to the inmates.

It is earnestly to be hoped that the Legislature of the State will carry on to perfection the noble work which it has commenced. Dr. Cooper demonstrates, in his report, that the asylum will be a great saving in money to the State. It shows, beyond all possibility of cavil, that the lunatics and idiots can be kept in a public institution at a much smaller cost than is now paid to individuals for their maintenance by the commonwealth. And in connexion with this exhibit, it presents a picture of human wretchedness, springing unavoidably out of the system of committing to individuals without responsibility the guardianship of the insane, at which the stomach, as well as the heart, turns sick.

The Lunatic Asylum of Georgia is situated within two miles of Milledgeville, of which it commands a picturesque view, in a district of country represented as being healthy, and within a few miles of the great central rail road. During the year, beginning November, 1843, and ending November, 1844, it had thirty patients, of whom four were discharged, three eloped, and one died. The cost of maintaining these, together with a steward and a matron, equal to nineteen persons for an entire year, was $450.

Dr. Cooper subjoins to his report a condensed history of all the cases in the asylum. We have room to notice but a single one of these, the particulars of which are extraordinary. A convict from the penitentiary was removed to the asylum in an idiotic condition, having been previously a lunatic. His appearance was eminently fatuous; from January, 1843, to May, 1844, he was never seen to take notice of a single incident, or heard to speak a syllable, never ate or drank unless food was given to him, never changed his place unless required to do so, and gave no heed to the calls of nature in other respects. Dr. Cooper on one occasion watched him for two hours without witnessing the slightest muscular movement of his face, even so much as the winking of his eyes. The slobber driveled from his mouth, and sometimes collected in his throat until he was threatened with suffocation. His abdomen swelled, showing signs of dropsy, and his lower extremities became œdematous. For these symptoms Dr. Cooper gave him hydragogue cathartics, and after their operation, and the removal of the hydropic appearances, he showed some signs of returning consciousness, scratching his leg, and, after a time, asking for water and food. He continued slowly to improve, began to walk, and to obey the calls of nature voluntarily, and by the first of September was so far recovered, that he was able to effect his escape from the asylum, and to make his way on foot to his mother's house, a distance of one hundred and fifty miles.

This report is not carefully written, and is worse printed; but it presents to the people of Georgia a mass of facts in which they must feel the deepest interest. And not to the people of Georgia alone. It has interest for all who dwell in the Southern States. The institution whose brief history it records is another added to the asylums for this class of our afflicted, and is opened on terms so low that many, who have not heretofore sent their unfortunate friends abroad, will be able to avail themselves of its shelter.

